# 988. Is There Value of Infectious Diseases Consultation in Candidemia? A Single Center Retrospective Review From 2016-2019

**DOI:** 10.1093/ofid/ofab466.1182

**Published:** 2021-12-04

**Authors:** Jonathan H Ryder, Trevor C Van Schooneveld, Trevor C Van Schooneveld, Erica J Stohs

**Affiliations:** University of Nebraska Medical Center, Omaha, Nebraska

## Abstract

**Background:**

Candidemia is the second most common cause of healthcare-associated bloodstream infections in the US with mortality of approximately 25%. Studies demonstrate lower candidemia mortality with infectious diseases consultation (IDC). We evaluated effects of IDC on mortality and guideline-adherence at our institution to determine if mandatory IDC was warranted.

**Methods:**

We retrospectively reviewed adults hospitalized with candidemia (≥ 1 blood culture positive for *Candida*) between 1/1/2016-12/31/2019. Exclusion criteria included age < 19 years, polymicrobial blood culture, or death or hospice within 48 hours. Primary outcome was all-cause 30-day mortality. Secondary outcomes included guideline-adherence and treatment choice. Guideline-adherence was assessed with a modified EQUAL Candida score (Table 1). Descriptive statistics were performed.

Table 1. Original vs Modified EQUAL Candida Score

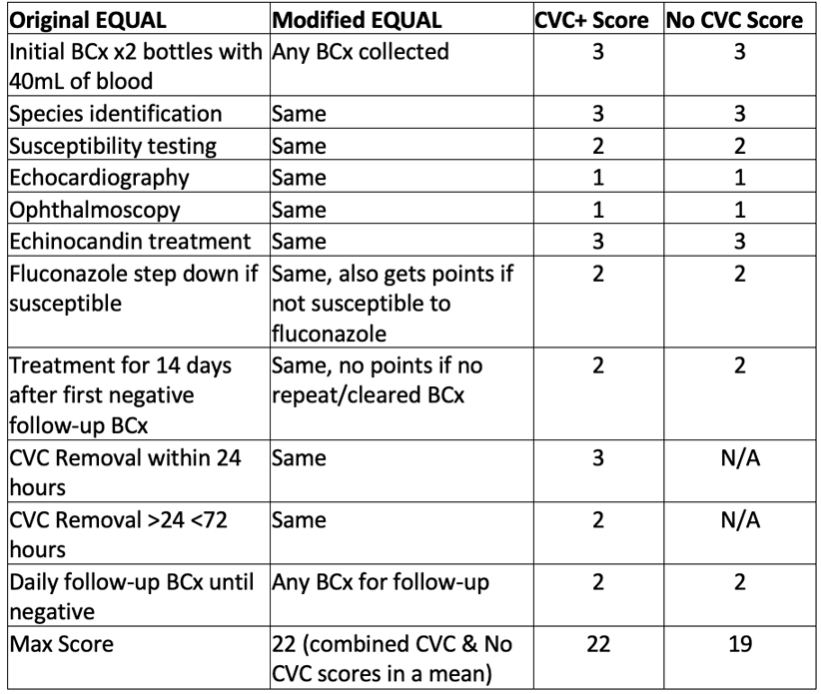

Abbreviations. CVC: central venous catheter, BCx: blood culture

**Results:**

Of 187 patients reviewed, 92 episodes of candidemia with 94 species of *Candida* were included. Patient characteristics are shown in Table 2. Central venous catheters (CVCs) were present in 66 (71.7%) patients and were the most common infection source (N=38 [41.3%]) followed by intra-abdominal (N=23 [25%]). The most isolated species were *Candida glabrata* (40/94 [42.6%]) and *C. albicans/dublienensis* (35/94 [37.2%]). 30-day mortality was 21.7%. IDC was performed in 84 (91.3%) cases. Outcomes are in Table 3. Mortality was not different between IDC vs no IDC (18 [21.4%] vs 2 [25%]); other comparisons were numerically different but not significant: repeat blood culture (98.8% vs 87.5%), echocardiography (70.2% vs 50%), CVC removal (91.7% vs 83.3%), and initial treatment echinocandin (67.9% vs 50%). All patients received antifungal therapy. IDC resulted in more ophthalmology consultations (77.4% vs 12.5%, p< 0.01). Mean modified EQUAL Candida score was higher with IDC (17.4 vs 13.9, p< 0.01).

Table 2. Patient Characteristics

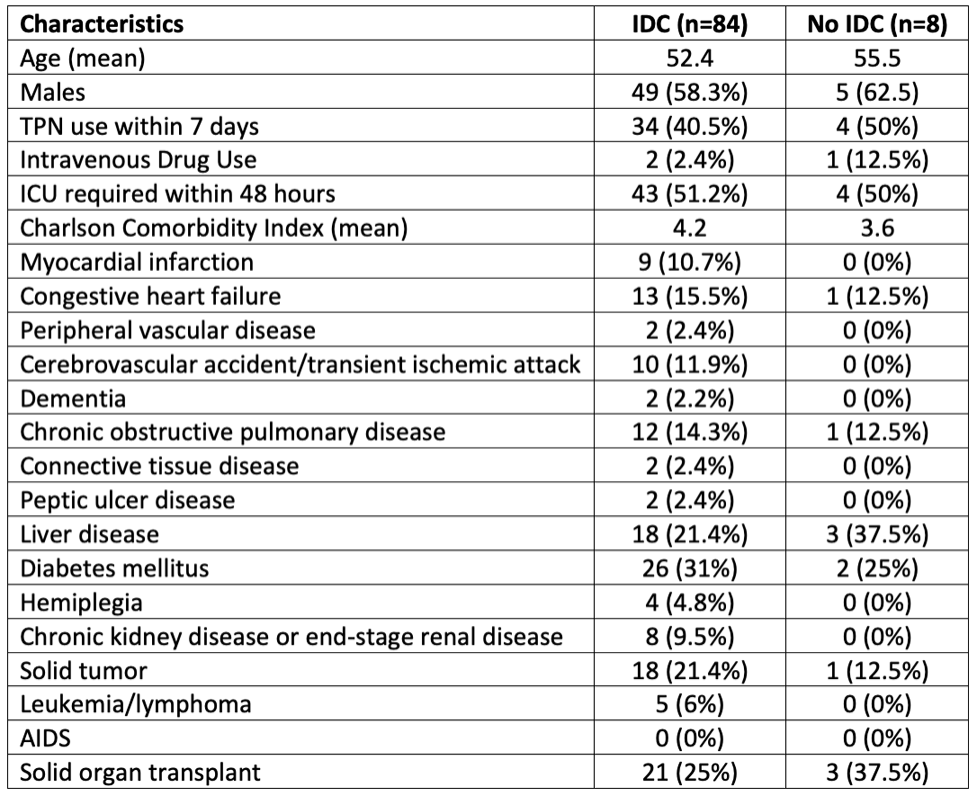

Abbreviations. TPN: total parenteral nutrition, ICU: intensive care unit, AIDS: acquired immunodeficiency syndrome

Table 3. Outcomes

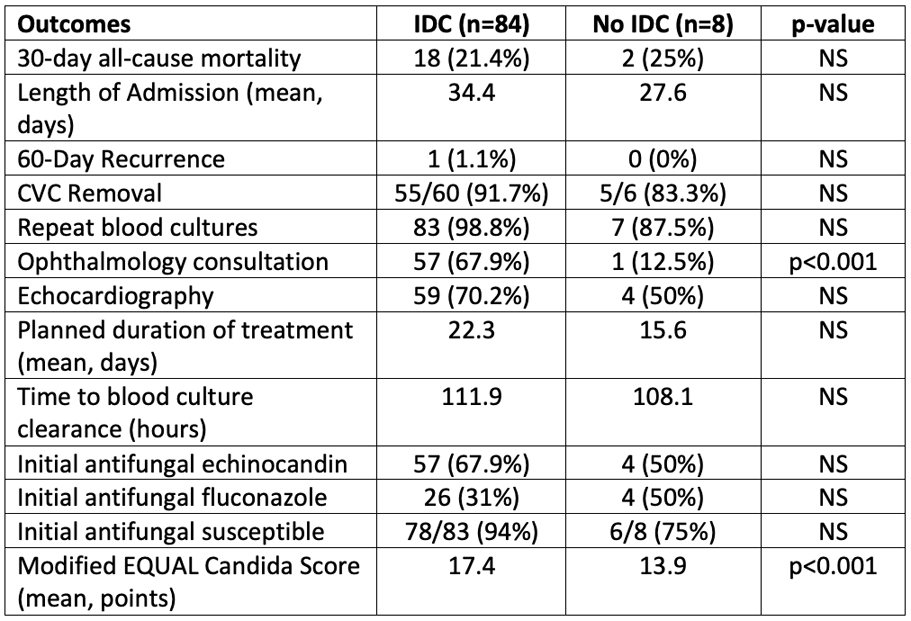

Abbreviations. NS: non-significant, CVC: central venous catheter

**Conclusion:**

IDC was common in candidemic patients and not associated with significant differences in outcomes. Current antimicrobial stewardship and consultation practices at our center do not warrant mandated IDC for candidemia.

**Disclosures:**

**Trevor C. Van Schooneveld, MD, FACP**, BioFire (Individual(s) Involved: Self): Consultant, Scientific Research Study Investigator; Insmed (Individual(s) Involved: Self): Scientific Research Study Investigator; Merck (Individual(s) Involved: Self): Scientific Research Study Investigator; Rebiotix (Individual(s) Involved: Self): Scientific Research Study Investigator

